# “It Was Traumatizing, Because It Makes You Feel Like You Are Not Right”: 2S/LGBTQIA+ Survivors’ Experiences Accessing Care for Intimate Partner Violence-Caused Brain Injury

**DOI:** 10.3390/healthcare14080997

**Published:** 2026-04-10

**Authors:** Emily Chisholm, Tori N. Stranges

**Affiliations:** 1 Department of History and Sociology, University of British Columbia–Okanagan, Kelowna, BC V1V 1V7, Canada; 2 School of Health and Exercise Sciences, University of British Columbia–Okanagan, Kelowna, BC V1V 1V7, Canada

**Keywords:** 2S/LGBTQIA+, intimate partner violence, brain injury, stigma, minority stress, help-seeking

## Abstract

2S/LGBTQIA+ survivors of intimate partner violence (IPV) face multiple, intersecting barriers to accessing care, yet little is known about how these barriers are shaped by IPV-caused brain injury (IPV-BI). Background/Objectives: This study aimed to explore how stigma and institutional trust influence 2S/LGBTQIA+ survivors’ experiences of help-seeking following IPV-BI. Guided by a Community Advisory Board, four semi-structured focus groups were conducted with 29 2S/LGBTQIA+ IPV-BI survivors. Methods: Reflexive thematic analysis was used to examine participants’ help-seeking accounts, with attention to minority stress and intersecting stigmas related to IPV, BI, and 2S/LGBTQIA+ identity. Results: The findings indicate that survivors navigated compounded stigmas that limited access to safe, affirming services and heightened vulnerability during help-seeking. Institutional trust was central to participants’ decisions to disclose sensitive information and engage in care, with confidentiality emerging as a critical determinant of perceived safety. Participants described negotiating disclosure, anticipating discrimination, and avoiding services when systems were perceived as unsafe or unresponsive. Conclusions: These findings highlight the need for service systems to integrate IPV-BI into screening and support protocols, provide training on the intersections of IPV, BI, and 2S/LGBTQIA+ identities, and centre confidentiality as a condition for trust and access, ultimately fostering safer, more responsive systems of care.

## 1. Introduction

Intimate partner violence (IPV) is a pervasive public health epidemic with long-term physical, emotional, and cognitive consequences. One of the lesser-recognized outcomes of IPV is brain injury (BI), often resulting from physical violence, sexual violence, and/or non-fatal strangulation [1,2,3,4]. These experiences can lead to lasting impairments in cognition, memory, attention, emotional regulation, and daily functioning [5,6,7,8]. Despite growing recognition of IPV-caused BI (IPV-BI) in research and practice, survivors who identify as Two-Spirit, lesbian, gay, bisexual, transgender, queer, intersex, asexual and/or other sexually and gender-diverse identities (2S/LGBTQIA+) remain critically underrepresented.

Existing approaches to understanding and supporting survivors of IPV-BI have largely reflected heteronormative, cisnormative, and settler colonial assumptions about relationships, gender, and help-seeking [9,10]. As a result, there is limited research examining how IPV-BI manifests within 2S/LGBTQIA+ communities. Distinct injury-related characteristics specific to this population are not yet well established. Rather, emerging work suggests that the context in which IPV-BI occurs may shape how injuries are experienced, recognized, and responded to. For 2S/LGBTQIA+ survivors of IPV-BI, engagement with systems of care (interconnected network of formal and informal services, institutions, and supports that collectively respond to survivors of IPV-BI) often unfolds within contexts marked by stigma, discrimination, and unequal power relations [11,12,13,14,15,16,17,18]. Discrimination against 2S/LGBTQIA+ people manifests as heterosexism and cisnormativity in forms, policies/protocols, medical records, and physical environments, further restricting access to inclusive and effective care [18,19,20,21,22]. 2S/LGBTQIA+ people report high rates of experiencing harsh or abusive language and being refused care, with trans patients facing particularly high levels of discrimination [18,19,23,24]. Service providers’ assumptions of heterosexuality and reliance on heteronormative frameworks further undermine survivors’ sense of safety and trust when seeking IPV and BI care [18,24,25,26,27].

Stigma and diminished institutional trust are well-documented barriers for 2S/LGBTQIA+ people accessing IPV-related services [9,10,14,28,29,30]. Stigma operates through social and institutional mechanisms that label, stereotype, devalue, and discriminate, producing and reinforcing status loss and power differentials [31]. Institutional trust, understood as the belief that systems are reliable, fair, and competent, is shaped through lived experience and broader perceptions of institutional norms. Institutional trust often eroded when individuals encounter stigmatizing, discriminatory, or inadequate responses, resulting in institutional betrayal and subsequent disengagement from services [28,30,32,33,34]. Foundational scholarship conceptualizes stigma as both interactional and structural, enacted and managed through identity processes such as concealment and disclosure [35], and simultaneously produced through broader regimes of power, knowledge, and normativity that define deviance and legitimacy [36,37]. For individuals with concealable stigmatized identities, including many 2S/LGBTQIA+ IPV-BI survivors, disclosure requires ongoing evaluation of social context due to anticipated discrimination [33,38,39]. Anticipated stigma, expectations of rejection or mistreatment upon disclosure, has been linked to increased psychological distress, reduced help-seeking, and institutional mistrust [40,41,42,43]. In the context of 2S/LGBTQIA+ IPV-BI, structural stigma further marginalizes survivors by centring white, heterosexual, cisgender narratives of violence, limiting institutional recognition, and constraining access to appropriately equipped services [13,15,44,45].

Minority Stress Theory (MST) provides a useful theoretical framework for understanding how these barriers accumulate and shape 2S/LGBTQIA+ survivors’ experiences of IPV-BI and help-seeking [46,47]. MST posits that individuals from structurally marginalized sexual and gender minority groups are exposed to chronic, socially produced stressors that operate in addition to general life stressors, and are rooted in stigma, discrimination, and systemic inequities [20,46,47]. Operating on multiple levels, these stressors are conceptualized as distal stressors, such as institutional discrimination and violence, and proximal stressors, including expectations of rejection, identity concealment, and internalized stigma [46,48,49]. Within systems of care, stress processes can manifest as heightened vigilance, mistrust, and avoidance of care, particularly when prior interactions have been experienced as unsafe, dismissive, or harmful [32,33,34,46]. For 2S/LGBTQIA+ survivors of IPV-BI, many of whom navigate intersecting forms of marginalization related to gender identity, sexual orientation, race, disability, and class, minority stress offers a critical lens for understanding how systemic conditions shape both access to care and survivors’ sense of safety and trust within systems of care.

Existing research on IPV-BI emphasizes that addressing gaps in education and recognition, as well as improving the management, support, and prevention of IPV-BI, necessitates the development of context-specific and comprehensive systems of care [1,50]. Such systems must be specialized, interdisciplinary, and holistic, and include integrated navigational and advocacy supports to effectively respond to survivors’ needs [1]. However, the limited inclusion of 2S/LGBTQIA+ survivors’ perspectives within IPV-BI research limits the ability of existing models to adequately account for the realities of accessing care within the 2S/LGBTQIA+ community.

To address this gap, the current paper presents findings from a series of focus groups with 2S/LGBTQIA+ individuals who have experienced IPV-BI. By centring survivors’ narratives, this research sought to better understand how 2S/LGBTQIA+ survivors navigated systems of care, what barriers they faced in accessing meaningful support, and what practices contribute to (or undermine) healing. In doing so, this study contributes critical conceptual insights to IPV-BI scholarship and contributes to the development of care systems that are trauma-informed, queer- and trans-affirming, anti-colonial, and structurally responsive to the realities of survivors’ lives.

## 2. Materials and Methods

This study was guided by a Community Advisory Board composed of service providers and 2S/LGBTQIA+ advocates with lived experience of IPV-BI. The Community Advisory Board provided input throughout the research process, including study design, recruitment strategies, the development of a trauma-informed focus group guide, and review of findings. Their involvement ensured that the research was grounded in community priorities, relevant to the 2S/LGBTQIA+ community, and attentive to relational accountability.

Four semi-structured focus groups were completed in March of 2025, with 5–9 participants per group and durations ranging from 58 min to 95 min (FG1: 1:35:04, n = 7; FG2: 1:12:28, n = 9; FG3: 1:04:47, n = 8; FG4: 0:58:06, n = 5). The focus groups were facilitated by the second author (TNS) who brings over 10 years of experience working in BI and 5 years of research focused on IPV-BI in 2S/LGBTQIA+ relationships, with co-facilitation provided by the first author (EC) who worked as a research assistant on the project. Both facilitators brought relevant background and lived/community knowledge that informed engagement with participants.

Focus groups allowed for the exchange of insights, perspectives, and questions [51]. This method was chosen for this community-oriented study because it provided space for mutual support, enhanced reflexivity, and empowered participants by centring their voices in knowledge production [52]. Focus groups were especially well suited to research stigmatized experiences, like IPV-BI, as they allowed 2S/LGBTQIA+ participants to articulate perspectives that may diverge from dominant narratives while engaging in dialogue that affirms their shared experiences. Intersectional approaches to qualitative research emphasize that such engagement is critical to ensuring findings are responsive to the needs of marginalized populations [53].

In addition to capturing rich interpersonal dynamics that may not emerge in individual interviews [52], focus groups are frequently used in social action research because they generate structural rather than individual explanations of social issues. For this study, semi-structured focus groups were chosen to facilitate collective dialogue, support relational accountability with 2S/LGBTQIA+ communities, and ensure the production of community-grounded knowledge. This approach aligned with the project’s objective of generating competent and responsive evidence to inform service provision for 2S/LGBTQIA+ survivors of IPV-BI.

Data was analyzed using reflexive thematic analysis [54]. Analysis was iterative and inductive, beginning with familiarization through repeated reading of transcripts, generating initial codes, and then grouping codes into potential themes [54,55,56]. Consistent with a reflexive approach, the research team engaged in ongoing critical reflection on their positionality, with interpretations shaped by the second author’s expertise in IPV-BI and 2S/LGBTQIA+ health and the co-researcher’s lived/community perspective, to support reflexivity and contextual grounding of the analysis. Themes were reviewed, defined, and refined through team discussions to ensure interpretations reflected community perspectives and remained contextually grounded. NVivo (14.24.0; Lumivero, Denver, CO, USA) software was used to organize data and facilitate analysis.

### 2.1. Recruitment

Participants were recruited as part of a larger community-based research project exploring the experiences of 2S/LGBTQIA+ individuals who had experienced IPV-BI in a 2S/LGBTQIA+ relationship. Recruitment materials were shared through provincial community organizations, social media, and targeted outreach to networks serving 2S/LGBTQIA+ communities. Interested individuals were directed to an online eligibility screener survey via QR code or direct link, which served multiple purposes including confirming participation eligibility, collecting basic demographic information, and screening for a potential IPV-BI.

The screener included closed- and open-ended demographic questions (e.g., gender identity, sexual orientation, age, and race) and a modified version of the HELPS tool [57], an evidence-based IPV-BI screening questionnaire that was adapted for online use. Adaptations included structuring items for self-administration (e.g., yes/no and brief descriptive responses) without facilitator support. The modified HELPS tool included questions about potential head injuries sustained in an intimate relationship, loss of consciousness, experiences of being “choked out”, symptoms associated with an IPV-BI, and other forms of physical violence often associated with IPV-BI. A positive screen was defined as endorsement of a head injury event in the context of IPV with or without loss of consciousness and/or the presence of acute or persistent post-injury symptoms. The tool was carefully adapted to be trauma-informed for 2S/LGBTQIA+ individuals, concise, and appropriate for self-administration in an online format.

Participants who met eligibility criteria (i.e., self-identified as 2S/LGBTQIA+, had experienced IPV, and screened positive for potential BI) and indicated interest in participating in a focus group were contacted via email. Participants were provided a detailed consent letter and information sheet outlining the study’s purpose, participation expectations, confidentiality considerations, and support resources. Eligible and consenting participants were then scheduled into one of four semi-structured focus groups held online via Zoom. To protect confidentiality and create a safe environment, participants were provided with community guidelines that emphasized respect, supportive engagement, and privacy. These guidelines included encouragement to mute microphones when not speaking, optional camera use, appropriate use of the chat function, and reminders that what is shared in the group remains confidential. Facilitators actively monitored the sessions and provided support through a designated Zoom support space if needed.

### 2.2. Data Collection and Analysis

Detailed characteristics of the sample are presented in Table 1. Participants were able to select multiple categories for sexual orientation and gender identity, while determinants of health and types of violence experienced were assessed using yes/no questions, with definitions provided for each type of violence. The average age of participants was 28.69 years (age 25–30, SD = 2.29). The sample reflected substantial diversity in sexual orientation and gender identity, with participants identifying across gay, lesbian, bisexual/pansexual, and queer orientations. Gender diversity was prominent, particularly among genderqueer and transgender participants, alongside non-binary and Two-Spirit identities. Over half of participants also identified as racialized or a visible minority, underscoring the intersecting social locations represented in the sample (see Table 1).

All participants (n = 29) reported experiencing physical, psychological, and financial abuse. All but one participant (n = 28) reported sexual and identity-based abuse. BI was widespread as 100% (n = 29) of participants reported being hit on the head by a partner, 97% (n = 28) reported loss of consciousness as a result of the physical violence they experienced, 97% (n = 28) reported lasting impacts on cognitive, emotional, or physical function, and 93% (n = 27) required medical attention. Of note, participant quotations were maintained verbatim to avoid imposing interpretive assumptions about sex or gender. As a result, some quotations reflect participants’ own conflation or interchangeable use of sex- and gender-related terms.

## 3. Results

Two main interconnected themes emerged from the focus group data including stigma and the formation of institutional trust. For 2S/LGBTQIA+ survivors of IPV-BI, stigma operated on multiple, overlapping levels. The experience of stigma shaped 2S/LGBTQIA+ survivors’ experiences within systems of care by informing institutional trust. Participants’ trust or mistrust in systems of care was actively constructed through encounters that either reinforced or disrupted stigma during their help-seeking efforts. When 2S/LGBTQIA+ survivors entered care systems, they had to weigh the risk of disbelief, discrimination, or breaches of confidentiality as they actively negotiated whether, when, and with whom to disclose information about their 2S/LGBTQIA+ identity, BI, and IPV experience. Confidentiality functioned as a central mechanism through which institutional trust was negotiated, making it a tipping point for whether survivors engaged or disengaged with systems of care.

### 3.1. Theme 1: Stigma

Participants spoke about navigating three overlapping forms of stigma: (1) BI stigma and the cognitive challenges associated with the injury, (2) IPV stigma and the risk of disbelief, and (3) 2S/LGBTQIA+ identity-based stigma and erasure. These systems did not operate independently; rather, they intersected and compounded, creating complex barriers that shaped 2S/LGBTQIA+ survivors’ help-seeking experiences, often exacerbating vulnerability, limiting access to appropriate supports, and reinforcing structural inequities. Participants described the constant effort of monitoring their safety and calculating risks as emotionally taxing; as Dylan, a 27-year-old non-binary, queer person shared, “navigating services felt overwhelming, and not all spaces were inclusive.” To protect themselves, survivors often choose to disengage from care systems that were deemed unsafe and untrustworthy. For many, this decision was not about a lack of need, but about anticipating harm based on prior experiences of stigma, dismissal, or discrimination within service environments. As Andres, a 32-year-old transgender (trans), gay man shared, “There is a fear of discrimination when trying to access community programs. I feel I will be harassed by service providers, and this leads to reluctance in seeking help.”

Aligning with MST [46,47], the cumulative effect of these stigmas produced chronic stressors as 2S/LGBTQIA+ IPV-BI survivors endured sustained exposure to multiple sources of discrimination, increasing psychosocial strain and limiting their capacity to navigate fragmented care systems. As Cameron, a 32-year-old trans, bisexual man summarized:

“My brain injury experience felt unique due to my 2S/LGBTQ identity because I faced additional layers of stigma and misunderstanding. Navigating both the physical and cognitive challenges of brain injury while also dealing with discrimination or invisibility related to my sexual or gender identity made it harder to find the support I needed.”

Morgan, a 28-year-old trans, gay man shared similar reflections when he shared, “Lack of provider awareness about IPV-related brain injuries, and services that weren’t designed for people with cognitive challenges. Many programs also weren’t inclusive of my 2SLGBTQ identity, making it harder to find safe, affirming support.” These accounts underscored how stigma operated structurally within care systems, reinforcing minority stress and limiting options for support. Stigma was not experienced as a single barrier, but as an interlocking and cumulative process that shaped 2S/LGBTQIA+ IPV-BI survivors’ interactions with systems of care. The convergence of BI-related stigma, IPV-related disbelief, and identity-based erasure produced environments that survivors perceived as unsafe, cognitively inaccessible, and emotionally depleting.

#### 3.1.1. Brain Injury

Stigma surrounding BI constituted a significant barrier to care. Participants described challenges accessing services due to limited provider training and systemic inattention to IPV-BI, noting that many service providers lacked expertise in identifying or accommodating cognitive difficulties such as memory loss, fatigue, or processing challenges. As Noah, a 27-year-old cisgender, queer woman explained:

“I think cognitive difficulties, you know, like memory problems, attention deficits and ability to process issues, can make it hard for individuals to try to navigate the healthcare system. Trying to schedule appointments, or even understand their own needs. So, also, it’s hard for someone like me [a 2S/LGBTQIA+ person who experienced an IPV-BI] to try and, you know, do things at an appropriate time.”

These insights highlighted how systems of care were not designed to accommodate the cognitive deficits associated with IPV-BI. Similarly, options for integrated support remained scarce. As Taylor, a 31-year-old trans, lesbian woman explained, “there was little recognition of how IPV caused my brain injury, leading to misdiagnosis and a lack of tailored resources for my recovery.” Other participants experienced similar obstacles. For instance, Evan, a 29-year-old non-binary, bisexual person described their experience going to the hospital to seek care following IPV-BI:

“I didn’t have anyone’s support at that moment because everybody thought that the fault was from me, and it was difficult to get to the hospital […] It’s better when someone has bleeding on the outside rather than having internal bleeding in the brain.”

Both Evan and Taylor faced challenges with having their BI adequately attended to due to a lack of understanding or support surrounding the connection between IPV and BI. Their experiences exemplify the compounding forms of stigma that amplify existing barriers to accessing care for 2S/LGBTQIA+ survivors of IPV-BI.

#### 3.1.2. IPV

Participants described how IPV-related stigma shaped both their decisions to seek help and their encounters with systems of care. Many anticipated disbelief, judgement, or minimization of their experiences, particularly when their identities, bodies, or relationships did not align with dominant, heteronormative understandings of IPV. These expectations of stigma often delayed help-seeking and heightened participants’ vulnerability, as survivors carefully weighed the risks of disclosure against the possibility of receiving support.

Several participants described internalized shame and fear prior to seeking care, reflecting broader social narratives that render IPV illegitimate outside of cisgender, heteronormative frameworks. Jordan, a 25-year-old trans, lesbian woman described the emotional labour involved in their decision of whether to seek mental health support:

“At first, I was scared to seek mental health support since my situation was complex. And, I was also ashamed of saying that my injuries were because of IPV. But after gaining confidence to express myself I received a cold response from the mental health supporters.”

Jordan further explained that their experience of dismissal was shaped by how their body and gender presentation were interpreted by service providers, “I have a big body which looks more masculine yet I’m a female. So, it was hard for me to convince them that I was involved in IPV.”

For Jordan, and for many others, IPV-related stigma intersected with identity-based stigma, producing compounded barriers to care. When violence itself was questioned or minimized, attempts to seek help were hindered by a lack of recognition that the harm was real and deserving of support. Participants described how systems of care reinforced these stigmatizing dynamics. Morgan, a 28-year-old trans, gay man touched on their experience of anticipated stigma when they noted how IPV programs limited access to safe spaces: “many [IPV] shelters and [IPV] programs are not gender-inclusive, making access to safe spaces difficult. Fear of being outed or not taken seriously also creates additional barriers.” Jaden, a 25-year-old non-binary, bisexual person similarly described interactions with service providers that felt accusatory and delegitimizing:

“The challenges I did face were going to access a service provider who was kind of asking me some questions like, “How did it happen? What caused the fight?” … While I was trying to tell the story, I was kind of torn down … that’s when the trauma comes in, and the stigma.”

Rather than facilitating support, Jaden’s encounter reinforced perceptions that disclosure would lead to further harm. Charlie, a 28-year-old non-binary, lesbian person articulated how fear of discrimination and embarrassment shaped their reluctance to seek mental health support, “I feel that I may not be listened to, or I may be discriminated against at one point or the other … that challenge makes me feel really reluctant about it. I feel it’s not really necessary [to seek support].”

Across these accounts, anticipated IPV-related stigma functioned as a powerful deterrent to help-seeking. Participants’ expectations of disbelief, judgement, or rejection were shaped by prior experiences within systems of care, as well as by dominant heteronormative narratives of IPV that contribute to a social erasure of 2S/LGBTQIA+ survivors of IPV and IPV-BI.

#### 3.1.3. 2S/LGBTQIA+ Identity

Participants described pervasive experiences of identity-based stigma when attempting to access services, including refusal of services, misgendering, inappropriate questioning, and a lack of affirmative care practices from providers. Collectively, these accounts illustrate how systems of care often operate with a narrow and exclusionary understanding of IPV which fails to attend to the multiplicity and complexity of experiences of IPV within 2S/LGBTQIA+ communities. Multiple participants reported being denied services outright on the basis of gender. Andres, a 32-year-old trans, gay man described being turned away from domestic violence services that framed IPV as an issue affecting only cisgender women:

“The domestic violence services I spoke to stated that they only provide services to women, not men. They believe that men cannot be the victim, only women. I believe if this discrimination or favoritism is removed, it will help others to open up to them.”

Exclusionary practices reinforced participants’ perceptions that systems of care were not designed to recognize or support 2S/LGBTQIA+ survivors, particularly those whose experiences, identities, and/or orientations deviated from cisnormative and heteronormative assumptions.

Misgendering and incorrect assumptions about identity were also frequently cited and described as deeply harmful. Sky, a 27-year-old trans, lesbian woman recounted being addressed with incorrect pronouns by care providers, characterizing the experience as overtly discriminatory and emotionally distressing. They shared, “it was traumatizing, because it makes you feel like you are not right. Like you are not doing the right thing.” Others similarly described having their identities assumed without consent, further undermining their sense of safety and legitimacy. Robin, a 29-year-old Two-Spirit, trans, gay woman shared, “Personally, my identity has always influenced how people treat me especially when I’m interacting with male gender, and they start thinking I’m male which really hurts to start explaining that I’m female by gender.” Participants also reported experiences of inappropriate or invasive questioning, as well as breaches of confidentiality. Hayden, a 32-year-old trans, bisexual man described encounters in which care providers asked about, “someone’s sexual orientation, gender identity or IPV experience without consent or sensitivity.” Cameron, a 32-year-old trans, bisexual man similarly noted, “in some situations, my identity or situation was assumed, and information was shared without my consent, leaving me vulnerable.” Difficulties accessing affirming and competent mental healthcare further compounded these challenges. Dylan, a 27-year-old non-binary, queer person described struggling to find a provider who could adequately support 2S/LGBTQIA+ clients, “because most times when I want to narrate my experience, it turns out that they mix up the pronouns and it makes my story cranked up.” Jamal, a 28-year-old trans, bisexual man echoed these experiences more broadly, stating, “I had bad experiences with discrimination, misgendering, or unwelcoming environments in shelters and healthcare.”

Beyond direct experiences of discrimination, participants described anticipated stigma as a significant barrier to seeking care. Prior to accessing services, many carefully weighed the risks of disclosure and expressed fear of judgement, dismissal, or mistreatment. When asked about barriers unique to 2S/LGBTQIA+ survivors of IPV-BI, Andres, a 32-year-old trans, gay man shared, “Fear of discrimination is a challenge that I face, this is what prevents me from seeking help”. Rowan, a 30-year-old cisgender, lesbian woman similarly noted, “I face provider bias from healthcare providers […] Sometimes I’m even scared of going out to get medical attention.” Riley, a 25-year-old trans, gay man reflected, “one of the challenges that I face is fear of discrimination. Since the survival of IPV, I feel that I may not be listened to, or I may be discriminated against at one point or the other.” Sky, a 27-year-old trans, lesbian woman further described how fear of disclosure constrained their willingness to seek support:

“Sometimes for me to just open up to someone, talk to someone whenever I want to access the care […] I have this feeling like, maybe if I share my story to anyone, people might possibly judge me so. I am so, so scared to open up to anyone at any moment.”

Taken together, these accounts demonstrate how stigma is enacted through institutional encounters that label, devalue, and delegitimize 2S/LGBTQIA+ survivors within systems of care. Both explicit and anticipated stigma [40,46] produced significant psychological distress and discouraged participants from engaging with systems of care. In addition to the physical and emotional harms associated with IPV and/or IPV-BI, participants experienced compounding harm through discrimination and stigma that targeted their gender identity and sexual orientation. Intersecting and compounding with experiences of IPV and BI stigmas, these identity-based exclusions reinforced structural barriers and eroded survivors’ willingness to seek support.

### 3.2. Theme 2: Institutional Trust

Institutional trust emerged as a key concept for understanding participants’ experiences of anticipated stigma when engaging with systems of care. Participants described how their trust in institutions shaped their expectations regarding whether or not institutions would fulfil their intended roles in responding to IPV-BI. For many 2S/LGBTQIA+ survivors, interactions with these systems were characterized by experiences of betrayal, exclusion, and unmet needs, which undermined trust and informed decisions about whether to seek support. As Cameron, a 32-year-old trans, bisexual man explained:

“As a 2SLGBTQ [IPV-BI] survivor, I face unique challenges like discrimination, lack of inclusive support, and the fear of outing. Access to safe housing and legal support that recognizes my identity is also limited, making it harder to escape abusive situations. Many providers aren’t fully understanding of my experiences, which creates mistrust.”

Similarly, Ryan, a 29-year-old Two-Spirit, non-binary, gay person highlighted, “To me it’s systemic discrimination and lack of competence in the sense that many healthcare providers lack knowledge and training to understand the specific needs and experiences of the people like me leading to inadequate and harmful care.” Together, Ryan and Cameron’s reflections illustrate that institutional trust is shaped through the interaction of provider practices, institutional policies, and survivors’ prior experiences of stigma and discrimination. Participants pointed to a pattern of doubt and mistrust towards the systems intended to provide care and support. Rather than reflecting individual, isolated encounters, this pattern of mistrust emerged as a collective response to systemic discrimination and recurring institutional failures across multiple care settings.

Institutional trust also influenced survivors’ willingness to engage with formal systems of care. Cameron, a 32-year-old trans, bisexual man contrasted experiences with institutional services and informal support networks, noting

“In my experience, personal communities have been more supportive than formal services. They offer emotional validation, practical help, and a sense of safety without the barriers like waitlists or stigma. Friends and family are more accessible and flexible, which made a huge difference when navigating tough times.”

Together, these accounts demonstrate that institutional trust functions as a foundational condition for accessing help-seeking among 2S/LGBTQIA+ survivors of IPV-BI. When systems of care are perceived as discriminatory, incompetent, or unsafe, survivors are less likely to engage or re-engage with formal services.

#### 3.2.1. Confidentiality

Confidentiality represented a core determinant of whether survivors felt safe engaging with systems of care. It was not perceived merely as a procedural requirement but as a prerequisite for safety and access. Many participants recounted breaches or perceived breaches of confidentiality, instances where personal information was shared without consent, unwanted questions were asked, or assumptions were made about identity or circumstance. As Cameron, a 32-year-old trans, bisexual man shared,

“My confidentiality was most at risk when I had to share personal information with providers who didn’t fully understand the importance of privacy or when I felt pressured to disclose details I wasn’t ready to share. In some situations, my identity or situation was assumed, and information was shared without my consent, leaving me vulnerable.”

These experiences often resulted in survivors disengaging from services and hesitating to approach others. For participants, confidentiality was more than a bureaucratic safeguard or an individual preference; it was an embodied form of trust that signals whether a system is safe. Participants emphasized that confidentiality must be both relational and institutional, requiring competent policies and provider training in how to gather these data in an appropriate way, while safeguarding patient privacy and confidentiality. When asked what service providers should do to ensure confidentiality is maintained, Cameron, a 32-year-old trans, bisexual man suggested:

“Service providers should have clear confidentiality policies, ensure staff are trained on privacy standards, and use secure systems for handling personal information. They should also communicate openly about what will and won’t be shared and gain explicit consent before disclosing any details. Creating a safe, trustworthy environment is key to maintaining confidentiality.”

Confidentiality emerged not just as a good practice, but as a prerequisite for safety and access. Providers who explicitly affirmed confidentiality, demonstrated knowledge of contextual norms, and respected these boundaries enhanced survivors’ sense of safety.

#### 3.2.2. Negotiating Disclosure

When 2S/LGBTQIA+ IPV-BI survivors share sensitive information, their decision is informed by relational and institutional trust, not just personal comfort. For 2S/LGBTQIA+ survivors of IPV-BI, disclosure is not a one-time act but a continuous process of negotiation. As Jordan, a 25-year-old trans, lesbian person explains, “As an LGBTQ member, we are sometimes choosy when seeking some of these services.” This “choosiness” refers to the ways that survivors constantly calibrate how much to reveal or conceal depending on context, relationship, and temporal needs. 2S/LGBTQIA+ survivors modulate disclosure of IPV and identity based on perceived safety and control within care encounters. Participants shared the acute attention they give to aspects of the social environment when negotiating disclosure in specific contexts. As Carter, a 30-year-old cisgender, lesbian woman shared,

“Personally, I feel like, you know, sharing this kind of information has to do with […] knowing the environment where I’m sharing the information. This has to be an environment with a good atmosphere, and I actually need to be safe.”

Further, Riley, a 25-year-old trans, gay man shared, “the body language of the service provider really matters as well. If I feel like the person is feeling kind of uncomfortable with what I’m saying, it’s going to weigh me down.” For 2S/LGBTQIA+ survivors, minority stress and fear of being outed or discriminated against add a further layer of risk when it comes to disclosure, making confidentiality and competent response crucial determinants of engagement. Jordan, a 25-year-old trans, lesbian woman shared:

“I would like the community leaders to know that as an LGBTQ member, sometimes it’s hard to open up on issues to do with traumatic experiences. So when the organizations that are supposed to give us a shoulder to lean on become judgmental, we get scared to seek any form of help.”

Jordan highlights that when institutions fail (through ignorance of IPV-BI, insensitive responses, or breaches of confidentiality), survivors experience institutional betrayal that both retraumatizes and discourages future help-seeking [34]. These statements underscore how disclosure decisions are deeply intertwined with institutional trust and perceptions of provider competence, empathy, and inclusivity. In contexts where confidentiality is respected and staff are educated on the intersecting systems of discrimination and community-specific needs, 2S/LGBTQIA+ survivors of IPV-BI are more likely to perceive the system as trustworthy and re-engage in care.

## 4. Discussion

This study found that when navigating systems of care, 2S/LGTBQIA+ survivors of IPV-BI encountered intersecting and compounding forms of stigma related to gender identity and/or sexual orientation, IPV, and BI that impacted their help-seeking experiences. These stigmatized encounters, alongside gaps in provider knowledge regarding IPV-BI, cognitive difficulties, and breaches of confidentiality, eroded 2S/LGBTQIA+ IPV-BI survivors’ trust in institutions and shaped their willingness to seek and engage with formal systems of care.

Consistent with foundational conceptualizations of stigma as both interactional and structural, these experiences were not limited to isolated incidents of discrimination but reflected broader regimes of normativity that determine whose victimization is legible, whose injuries are taken seriously, and whose identities are affirmed within institutional settings [31,35,58]. For 2S/LGBTQIA+ participants, stigma was enacted through dismissive provider interactions, misgendering, invasive questioning, as well as gendered shelter policies, non-inclusive service eligibility criteria, and the ways that systems of care failed to accommodate cognitive impairments associated with IPV-BI. 2S/LGBTQIA+ IPV-BI survivors described weighing the risks of discrimination, dismissal, or harassment against the potential benefits of support [40,59]. Thus, anticipated stigma may have also played a role in influencing decisions around identity and injury disclosure, concealment, and help-seeking.

For those navigating IPV-BI, difficulties in executive functioning, memory, attention, and information processing may have further complicated decisions around disclosure and help-seeking, intensifying survivors’ vulnerability [5,6,7]. Previous research shows that IPV-BI often goes unrecognized or is dismissed by providers, with IPV-BI survivors reporting that overlapping cognitive and emotional symptoms can compound barriers to accessing care and increase reliance on informal support networks [1,60,61,62]. These studies illustrate that survivors’ IPV-BI symptoms are not only neurologically disabling but also socially consequential. When providers lack training in IPV-BI, symptoms may be misinterpreted, misattributed, or missed altogether, minimizing survivors’ needs and further discouraging engagement with formal services.

In line with the existing literature, confidentiality represented a critical consideration through which 2S/LGBTQIA+ survivors of IPV-BI negotiated institutional trust [21,63]. Participants described heightened vigilance around disclosure and carefully weighing when, how, and with whom to share sensitive information about their IPV-BI. Consistent with Whitton et al. [17], who found that 2S/LGBTQIA+ IPV survivors often withheld information due to anticipated bias or discrimination, our findings also illustrated how breaches, or even perceived breaches, of confidentiality directly discouraged engagement with services. 2S/LGBTQIA+ IPV-BI survivors in our sample described experiences in which providers assumed identities, asked invasive questions without consent, or failed to safeguard personal information, reinforcing perceptions that systems were unsafe. These encounters may have compounded the challenges associated with managing cognitive impairments from BI, as difficulties with memory, processing, and self-advocacy amplified the risks of disclosure in unsafe care contexts [32,34]. These findings underscore the importance of integrating explicit confidentiality practices, trauma-informed approaches, and staff training into services that IPV-BI survivors access to create environments in which 2S/LGBTQIA+ survivors feel safe.

Consistent with MST’s emphasis on concealment vigilance as a proximal stress process [46], participants in this study described being “choosy” in their help-seeking practices. This selectivity may be understood as a protective or adaptive response to environments in which disclosure can carry material risks. These proximal stress processes appeared to emerge in the context of repeated exposure to distal stressors, including gender-segregated services, heteronormative assumptions about IPV, and a lack of providers who are equipped to support 2S/LGBTQIA+ care needs [46,64]. Within this context, distal stressors may represent institutionalized forms of exclusion that constrained IPV-BI survivors’ options for care prior to any direct interaction. Therefore, structural stigma may have not only produced interactional harm but also fostered anticipated stigma by signalling to participants that their identities and experiences were unlikely to be recognized as legitimate within systems of care [65]. For survivors navigating IPV-BI alongside the marginalization of their 2S/LGBTQIA+ identity, these layered stigmas may have compounded stress and further eroded trust in systems of care.

Institutional trust served as a key mechanism linking stigma, minority stress, and help-seeking behaviour. Consistent with the existing literature [17,33,34], participants’ trust in healthcare, legal, and social service institutions was constructed through cumulative encounters with providers, policies, institutional practices, and relations to societal norms and histories of exclusion. Experiences of discrimination, misrecognition, inadequate IPV-BI knowledge, and breaches of confidentiality functioned as forms of institutional betrayal that eroded trust and discouraged future engagement with formal systems of care. Wherein institutions failed to protect confidentiality, accommodate IPV-BI-related needs, or demonstrate affirming practices, 2S/LGBTQIA+ survivors in this sample disengaged, often turning instead to informal networks perceived as safer and more responsive.

The findings of this study highlight several actionable areas for improving services for 2S/LGBTQIA+ survivors of IPV-BI. Participants emphasized that stigma—related to BI, IPV, and 2S/LGBTQ+ identity—intersected to shape their experiences of institutional trust, influencing whether and how they sought care. To address these barriers, services should prioritize the creation of inclusive and affirming environments that explicitly recognize and accommodate the unique needs of 2S/LGBTQIA+ IPV-BI survivors [16]. Key practice implications include enhancing provider training on IPV and IPV-BI to ensure recognition of BI-related cognitive challenges (e.g., memory, attention, processing difficulties) and the complex ways these intersect with IPV-BI survivors’ identities. In addition, provider training should be explicitly grounded in the community-specific needs of 2S/LGBTQIA+ survivors and include critical engagement with the historical and ongoing experiences of stigma, exclusion, and institutional harm within systems of care. Such training should also address the consequences of these systemic injustices, including the production of minority stress and the resulting patterns of mistrust and disengagement from formal support systems. Participants’ accounts highlighted the importance of relational practices grounded in empathy, respect, and patience, as well as the need for providers to demonstrate an explicit awareness of the heightened risks, vulnerabilities, and potential consequences associated with seeking formal assistance for IPV. Additional priorities include implementing cognitively accessible pathways of care, such as simplified intake procedures, clear communication, and flexible appointment structures; strengthening confidentiality safeguards and trust to reduce fear of disclosure and anticipated stigma; and improving integration across healthcare, legal, and social service institutions to reduce fragmentation and enhance continuity of support. Future research and service development initiatives should also prioritize the meaningful involvement of 2S/LGBTQIA+ survivors in the design, evaluation, and governance of IPV-BI services. Supporting the production of community-grounded knowledge will be critical to addressing persistent service gaps and to informing the development of integrated, multidisciplinary systems of care that are responsive to survivors’ needs. Evaluating the effectiveness of these strategies in improving accessibility, trust, and outcomes for 2S/LGBTQIA+ survivors of IPV-BI represents an important direction for future research.

These findings underscore that institutional trust is inseparable from both relational practice and institutional procedures, particularly for survivors managing BI and navigating concealable stigmatized identities. Further, these findings provide a conceptual contribution to MST by illustrating that within IPV contexts, BI intensifies minority stress for 2S/LGBTQIA+ survivors. Echoing MST’s emphasis on chronic stress exposure [15,20,22,64], participants described the ongoing labour of monitoring safety, negotiating risks of disclosure, and managing stigma as exhausting and overwhelming. Symptoms of BI such as memory difficulties, processing challenges, fatigue, and emotional dysregulation interact with chronic stress to heighten distress and reduce survivors’ capacity to navigate already hostile or inaccessible systems of care. These insights emphasize that effective service design must consider not only the physical and cognitive sequelae of IPV-BI but also the intersecting minority stressors faced by 2S/LGBTQIA+ survivors, reinforcing the need for practice-informed, community-engaged approaches that integrate evidence-based strategies and culturally affirming care.

### Limitations

This study has several limitations. First, focus groups may have shaped what participants felt comfortable disclosing about highly stigmatized experiences such as IPV, IPV-BI, and sexual orientation or gender identity, and the findings therefore reflect collectively articulated experiences rather than exhaustive individual narratives. Second, because the semi-structured focus groups were conducted online via Zoom, participants’ engagement and attention to discussion topics may have varied in the virtual setting. Participants were recruited through community networks, which may overrepresent individuals with some level of connection to resources and underrepresent those who are more isolated or disengaged from systems of care. As experiencing IPV-BI was an inclusion criterion, the sample likely reflects individuals with more severe or readily identifiable BI experiences (e.g., loss of consciousness or seeking medical attention) which may have shaped the experiences captured in this study and limit transferability across the broader spectrum of 2S/LGBTQIA+ IPV-BI survivors. In addition, BI status was based on self-report screening rather than clinical diagnosis, consistent with IPV-BI research but limiting conclusions about injury severity. Further, the sample was relatively young and age-homogeneous (M = 28.69, SD = 2.29), which may limit the transferability of findings to older populations. Finally, while the sample reflected diversity across identities and social positions, the study was not designed for subgroup comparisons.

## 5. Conclusions

This study contributes critical insight into how stigma and institutional trust shape help-seeking among 2S/LGBTQIA+ survivors of IPV-BI. By centring the lived experiences of survivors, our findings demonstrate that stigma is not experienced as a singular barrier but as an interlocking and cumulative process operating across BI, IPV, and 2S/LGBTQIA+ identity. These intersecting stigmas function both interactionally and structurally, shaping how survivors are recognized, responded to, and either supported or excluded within systems of care. Institutional trust illustrates the connection between these processes: survivors’ decisions to engage, disengage, disclose, or conceal were actively negotiated in response to prior encounters with discrimination, inadequate IPV-BI knowledge, gendered service models, and breaches or ambiguities surrounding confidentiality.

This research underscores that access to care for 2S/LGBTQIA+ survivors of IPV-BI is fundamentally shaped by the conditions under which trust is built or broken. Institutional trust is not an abstract concept but a lived, relational, and structural phenomenon that determines whether survivors perceive systems of care as safe, legitimate, and responsive. Advancing responsive systems of care is therefore not only a matter of equity but a necessary condition for meaningful access to support for survivors navigating the compounded realities of IPV, BI, and minority stress.

## Figures and Tables

**Table 1:** Characteristics of the sample.

Sexual Orientation	n (%)
Gay	9 (31.0)
Lesbian	8 (27.6)
Bisexual/Pansexual	7 (24.1)
Queer	5 (17.2)
Gender Identity	n (%)
Genderqueer	10 (34.5)
Transgender Man	7 (24.1)
Two-Spirit	4 (13.8)
Non-Binary	4 (13.8)
Transgender Woman	3 (10.3)
Cisgender Woman	3 (10.3)
Cisgender Man	2 (7.0)
Determinants of Health	n (%)
Visible Minority or Racialized Group	16 (55.2)
Excluded Socioeconomic Group	10 (34.5)
Visible or Invisible Disability	9 (31.0)
Substance Use as Family or Individual	7 (24.1)
Housing Insecurity or Homelessness	5 (17.2)
First Nation, Metis, Inuit, Indigenous	4 (13.8)
Violence Experienced	n (%)
Physical	29 (100.0)
Psychological	29 (100.0)
Financial	29 (100.0)
Sexual	28 (96.6)
Identity-Based Abuse	28 (96.6)

Note: Participants were able to multiselect categories for gender identity and sexual orientation.

## Data Availability

The data presented in this study are available on request from the corresponding author due to confidentiality reasons.

## Abbreviations

The following abbreviations are used in this manuscript:

2S/LGBTQIA+	Two-Spirit, Lesbian, Gay, Bisexual, Transgender, Queer, Intersex, Asexual and/or other sexually and gender-diverse identities
IPV	Intimate Partner Violence
BI	Brain Injury
IPV-BI	Intimate Partner Violence-Caused Brain Injury
MST	Minority Stress Theory
Trans	Transgender
